# Staff preferences towards electronic data collection from a national take-home naloxone program: a cross-sectional study

**DOI:** 10.1186/s13011-022-00440-y

**Published:** 2022-02-16

**Authors:** Øystein Bruun Ericson, Desiree Eide, Philipp Lobmaier, Thomas Clausen

**Affiliations:** 1 The Norwegian Centre for Addiction Research, Building 45, Ullevål Hospital, Kirkeveien 166, 0450 Oslo, Norway; 2 King’s College London, National Addiction Centre, 4 Windsor Walk, London, SE58BB England

**Keywords:** Scaling-up health interventions, Staff preference, Buy-in, Implementation

## Abstract

**Background:**

During the scaling-up of a national Norwegian take-home naloxone (THN) program, data collection methods shifted from paper-based to electronic. The aim of this study was to explore staff preferences towards the shift in data collection.

**Methods:**

In January–February 2020, a survey was sent out via email to personnel involved with the THN program (*n* = 200). The survey included 17 questions, and covered staff demographics, experiences distributing THN, preferences towards data collection (both paper and electronically), and an open response section. Descriptive statistics were performed for the survey results. The open response section was recorded from each questionnaire and was coded into major themes by the authors.

**Results:**

In total, 122 staff completed the survey. Of these, 62% had experience with both electronic and paper-based forms, and there was a near unanimous preference towards electronic data collection over paper-based forms. From the free-text responses, staff found the electronic form to be a useful tool for conversation and overdose prevention education, and that the electronic form was easier to manage than the paper forms.

**Conclusion:**

The shift towards electronic data collection was necessary for the feasibility of the Norwegian national THN program. This study found that staff not only tolerated the shift, but in most cases preferred this organizational change.

## Background

Overdose mortality in Norway is approximately 260 per year, with opioids being involved in most cases [1]. In response to Norway’s overdose numbers, the Norwegian government launched a government-funded national overdose prevention strategy in 2014 [2]. One of the main interventions featured was a widespread take-home naloxone (THN) program. The program started as a pilot in the two most affected cities, but has since expanded to multiple municipalities [3].

Scaling-up of THN programs to reach relevant and sizeable populations of high-risk users is possible [4], however some studies have reported barriers related to implementation [5]. Specifically, these barriers include those related to workflow, staff roles, and responsibilities [5]. In other areas of public health, shifting administrative expectations have been associated with barriers related to both individual and organizational factors. This included issues with technical skills, lack of time, psychological and social factors, and how top-down implementation can potentially affect the process negatively, as it alienates the end-users from the intervention [6, 7].

In the Norwegian context, the scaling-up of the THN-program led to a wider reach of clients, but it came with implementation and operational challenges. One of the main challenges related to data collection. Program evaluation was explicitly included in the program development, and as such, data collection was an integral component. As the program expanded, collecting paper-based questionnaires and registration forms from several distribution sites across the country became unfeasible. Consequently, project management needed to shift the program’s data collection from paper forms to an electronic form.

Data collection was primarily conducted at low-threshold facilities, including drug consumption rooms and shelters [8]. Low-threshold facilities typically provides services for people with ‘complex and chaotic’ life styles [9]. These facilities are usually associated with harm reduction approaches, providing easy-access help to clients with severe social and health issues [9, 10]. When working with and conducting research with hard-to-reach groups in low-threshold settings, others have found barriers related to trust, data collection and registration procedures [10, 11]. Although electronic data collection has been used to document a variety of conditions in other areas of public health (primarily in clinical settings [7, 12]), the staff acceptability and preferences towards this method in a low-threshold setting has not previously been explored.

For the Norwegian THN program, it was necessary that the shift in data collection was acceptable to staff members implementing the program. The aim of this study was to evaluate staff preferences for electronic data collection for a large-scale THN program in a low threshold setting.

## Methods

### Study design

This study was a cross-sectional survey study exploring distribution staff preferences towards the shift from paper to electronic data collection in the Norwegian THN program.

### Setting

At the time of the study, THN was available at over 100 distribution sites throughout the country. Distribution sites included low-threshold facilities, rehabilitation centers, street outreach, medical clinics, and prisons. All staff members distributing naloxone through the THN program are required to complete the train-the-trainer-course developed by the THN program [13]. In addition to overdose prevention training and distributing naloxone, the trainer collects research data by interviewing clients on topics related to overdoses and naloxone use. The questionnaires are administered at the initial visit and for any subsequent refills.

The paper forms were either stored at the distribution sites until a project coordinator picked them up physically or returned by mail. The time needed for data entry of the paper forms also presented a delay in processing data. As the program expanded, the workload related to paperwork escalated proportionally. In 2018 the data collection shifted from paper forms to an electronic form to improve the processes.

The electronic form was developed in cooperation between peer advocacy groups, low-threshold facility staff members, and researchers. All of those that were involved with the forms (whether answering, administering, or analyzing the forms) were involved in the process. Others have found collaboration among different levels improves buy-in [14], and that improvements in data collection can benefit different groups of people [15].

The electronic form were located online via the THN program’s website. It was a simple open-source html page accessible to most browsers. The questionnaire was programmed to eliminate illogical pathways and consisted of checkboxes. Participant consent was collected electronically. The form was automatically stored in the University of Oslo’s safe storage for research solution.

### Participants

During a two-week period in January–February 2020, a survey was sent via email to personnel involved with the Norwegian THN program. Approximately 200 staff members received the survey directly by email. Recipients were encouraged to forward the survey to other colleagues and relevant personnel. A reminder was sent out one week later, and participants had two weeks to respond before the survey closed. Being a trained staff member at one of the distribution facilities with experience with both questionnaires were the eligibility criteria. Those who reported to only have experience with one of the questionnaires were excluded from the study.

### Survey

The survey included 12 questions, and covered staff demographics, experiences distributing THN, preferences towards data collection (both paper and electronically), and a free-text response section where participants could elaborate on the use of the electronic form and the program in general.

### Analysis

Frequency analyses were performed for the survey responses using SPSS statistics 27. The free-text response section was recorded from each questionnaire and was coded into major themes by the authors. The free-text response data provided insight and contextualization to each respondent’s preferences and experience relating to the Norwegian THN program and the data collection shift.

### Ethics

This study was approved by Norwegian Centre for Research Data (624040).

## Results

### Participants

In total, 122 staff members completed the questionnaire. Of those, 74 reported to have experience with both forms of data collection. Those who reported experience with just one of the forms were excluded from the study (*n* = 48). The majority (76%, *n* = 57) of respondents were women. All participants had worked at their current workplace for more than 12 months (*n* = 71) and their mean age was 40.8 years old. Participants were asked about previous naloxone trainer course, and most had taken their trainer course more than a year ago (70.3%, *n* = 52). The remaining completed the course less than 6 months ago (5.4%, *n* = 4), 6–12 months ago (10.8%, *n* = 8), were not able to recall (6.8%, *n* = 5) or reported never taking the course (6.8%, n = 5). Participants were also asked about their distribution activity, and most respondents reported distributing one or more naloxone kits in the past 6 months (85.1%, *n* = 63).

### Preferences towards data collection method

Participants were asked which type of form they preferred overall and on three distinct parameters: ease, speed, and accuracy. For nearly all parameters assessed, there was a clear preference for the electronic form (Table 1). The area of ‘overall preference’ showed the greatest preference towards the electronic form, with nearly all (94.6%, *n* = 70) preferring the electronic form. When asked if the electronic form affected the trainings, the majority (95.9%, *n* = 71) either found no difference or that it affected it in a positive way (Table 1).

**Table 1:** Survey results for data collection preference

	Number	Percent
**Which form is preferred?**	**74**	**100**
Electronic	70	94.6
Paper	2	2.7
No preference	2	2.7
**Which is easier to fill out?**	**74**	**100**
Electronic	70	94.6
Paper	2	2.7
No difference	2	2.7
**Which is faster to fill out?**	**74**	**100**
Electronic	65	87.8
Paper	3	4.0
No difference	5	6.8
Missing	1	1.3
**Which is more accurate?**	**74**	**100**
Electronic	42	56.8
Paper	5	6.8
No difference	27	36.5
**Does the electronic form affect overdose prevention training with the client?**	**74**	**100**
Yes, in a positive way	26	35.1
Yes, in a negative way	2	2.7
No difference	45	60.8
Missing	1	1.3

### Free-text response

In addition to the fixed-response survey questions, one free-text question was asked. Participants had the opportunity to give feedback related to the THN project in general, and regarding the data collection methods shift. Thirty-three participants elaborated on their preferences towards the shift to the electronic form in the free-text section. Two major themes emerged from this section.

The first was that the electronic form was a useful tool for conversation and overdose prevention education. Half of those who responded in the free text (51.5%, *n* = 17) elaborated on this topic. Responses included [translated from Norwegian]: “*The electronic form are a more natural way of obtaining information on the client’s drug habits through conversation.”* One respondent emphasized that, the electronic form had a positive impact on her relationship with the clients: “*It is a good way to explore and address traumatic experiences. It is a way to empower the client to talk about, and reflect upon experiences, and drug habits, with a focus on overdose risks. It also has a positive impact on our relationship with the client.”* This theme is further supported by the structured survey results in Table 1.

The second theme was about how the electronic form was easier to manage than the paper forms. Just over one-third (*n* = 12) of the answers categorized under this theme*: “With the paper forms I experienced that the client got a bit agitated seeing a stack of paper to fill out. This is not the case with the electronic form. Also, it is easier to use the THN instructional video as an educational tool when using the electronic form”.* One respondent found the electronic form to offer clarity: *“The questions are clear and specific, which encourages filling out the form.”*

## Discussion

This study found that overall, the shift to electronic form was not only tolerated, but in most cases, the shift was preferred. Specifically, staff found the electronic form to be a useful tool and were overall easier, faster, and more accurate to use than paper forms.

Implementation of modern digital interventions, despite its variety of advantages (such as optimizing and improving documentation), is also associated with individual barriers [6]. In this study, the new digital tool was preferred in most parameters. However whether such implementations have a positive effect or not, might depend on numerous factors, such as the context in which the technology is applied, its function, and how it was implemented [16]. The participants in our study reported a preference towards the electronic form, which may indicate improved buy-in. Further, from a function perspective, the electronic form was more ideal for researchers, easier and faster to use for the staff, and potentially less agitating for clients.

When implementing organizational changes in healthcare, promotion of a “shared vision” and buy-in is important [17]. This was supported in an assessment of the Norwegian THN program, where the researchers found that the adoption and uptake of the THN intervention was biggest where organizational (staff, leader and clients) buy-in was most prominent [13]. The social environment at the workplace should also be drawn to attention as a positive atmosphere might help improving reactions to digitalization [18]. When promoting the new electronic form, the researchers believed that the form would be easier to use, and that it would minimize workload related to the program’s data collection scheme. This is supported by the results of this study, with staff preferring the electronic form. The simplicity of the electronic form appeared to also offer an improved client visit, as one respondent reported that the clients don’t get agitated by “a stack of paper” when using the electronic form. One study monitoring pain found that electronic diaries were reliable, valid, and preferred by patients over paper diaries [19]. This study did not interview clients and their perspectives from the electronic form, although the speed and ease of it (as experienced by the staff) may give an indication that they may also prefer it.

For researchers, electronic data collection has been found by others to be more accurate and cost-effective [20–22]. From a research perspective, the real-time collection of electronic data into a database is faster than collecting and entering paper forms, and interactive validation (i.e., software that facilitates entry of ‘correct’ answers) allows for pre-defined ranges of expected values and logical skip patterns to be integrated in a way that paper forms cannot [9]. Several studies have also shown that electronic questionnaires are more accurate in terms of generating complete data and have fewer missing values than paper questionnaires [23–25].

### Limitations

The study assessed staff members’ preferences towards the data collection shift, and did not collect feedback from the clients, which could have given a broader perspective. In addition, our sample was a convenience sample and not necessarily a representative sample of the entire population. However, given that most had distributed naloxone within the last six months, we believe that we have reached relevant respondents nonetheless.

## Conclusions

Overall, our findings indicate acceptance towards the shift from paper forms to an electronic form for a national THN program, without loss of quality from the services provided to the end-users. Staff preferred the electronic form, which may translate to improved client interactions and data collected for research. The transition to the electronic form was necessary as the program expanded, as the physical collection and data entry for a large, nationwide program was not feasible. Others who are in a position to improve data collection practices, particularly with large-scale or widespread programs should consider the use of electronic forms. It is however important to consider buy-in from those collecting the data on a site-level to ensure that the format fits the setting, and to include client experiences as well.

## Data Availability

The datasets used and/or analyzed during the current study are available from the corresponding author on reasonable request.

## Abbreviation

THN: Take-home naloxone

## References

[1] Norwegian Institute of Public Health. Drug-related deaths 2020. [Translated from Norwegian]. Retrieved from www.fhi.no/nettpub/narkotikainorge/konsekvenser-av-narkotikabruk/narkotikautloste-dodsfall-2020/. Accessed 10 June 2021.

[2] Norwegian Directorate of Health (2014). [Translated from Norwegian] National Overdose Prevention Strategy 2014–2017 : “Sure you can be drug free, but first you must survive”.

[3] Norwegian Directorate of Health (2019). [Translated from Norwegian] National Overdose Prevention Strategy 2019–2022 : “Sure you can be drug free, but first you must survive”.

[4] Rowe C, Santos GM, Vittinghoff E, Wheeler E, Davidson P, Coffin PO (2015). Predictors of participant engagement and naloxone utilization in a community-based naloxone distribution program. Addiction..

[5] Drainoni M-L, Koppelman EA, Feldman JA, Walley AY, Mitchell PM, Ellison J (2016). Why is it so hard to implement change? A qualitative examination of barriers and facilitators to distribution of naloxone for overdose prevention in a safety net environment. BMC Res Notes.

[6] Boonstra A, Broekhuis M (2010). Barriers to the acceptance of electronic medical records by physicians from systematic review to taxonomy and interventions. BMC Health Serv Res.

[7] Zomahoun HTV, Ben Charif A, Freitas A, Garvelink MM, Menear M, Dugas M (2019). The pitfalls of scaling up evidence-based interventions in health. Glob Health Action.

[8] Madah-Amiri D, Clausen T, Lobmaier P (2017). Rapid widespread distribution of intranasal naloxone for overdose prevention. Drug Alcohol Depend.

[9] Morton S, O'Reilly L (2016). Community based low threshold substance use services: practitioner approaches and challenges.

[10] Edland-Gryt M, Skatvedt AH (2013). Thresholds in a low-threshold setting: an empirical study of barriers in a Centre for people with drug problems and mental health disorders. Int J Drug Policy.

[11] Bonevski B, Randell M, Paul C, Chapman K, Twyman L, Bryant J (2014). Reaching the hard-to-reach: a systematic review of strategies for improving health and medical research with socially disadvantaged groups. BMC Med Res Methodol.

[12] Jibb LA, Khan JS, Seth P, Lalloo C, Mulrooney L, Nicholson K (2020). Electronic data capture versus conventional data collection methods in clinical pain studies: systematic review and Meta-analysis. J Med Internet Res.

[13] Madah-Amiri D (2017). Opioid overdoses and overdose prevention: the establishment of take-home naloxone in Norway.

[14] Fleron B, Rasmussen R, Simonsen J, Hertzum M (2012). User participation in implementation. Proceedings of the 12th Participatory Design Conference: Exploratory Papers, Workshop Descriptions, Industry Cases - Volume 2.

[15] Holzinger A, Kosec P, Schwantzer G, Debevc M, Hofmann-Wellenhof R, Frühauf J (2011). Design and development of a mobile computer application to reengineer workflows in the hospital and the methodology to evaluate its effectiveness. J Biomed Inform.

[16] Bakke L, Garde AH, Nielsen MB, Sørensen K, Vleeshouwes J, Christensen JOF (2019). The influence of digitalization and new technologies on psychosocial work environment and employee health: a literature review.

[17] Applequist J, Miller-Day M, Cronholm PF, Gabbay RA, Bowen DS (2017). “In principle we have agreement, but in practice it is a bit more difficult”: obtaining organizational buy-in to patient-centered medical home transformation. Qual Health Res.

[18] Konttila J, Siira H, Kyngäs H, Lahtinen M, Elo S, Kääriäinen M (2019). Healthcare professionals’ competence in digitalisation: a systematic review. J Clin Nurs.

[19] Jamison RN, Raymond SA, Levine JG, Slawsby EA, Nedeljkovic SS, Katz NP (2001). Electronic diaries for monitoring chronic pain: 1-year validation study. Pain..

[20] Almeshari M, Khalifa M, El-Metwally A, Househ M, Alanazi A (2018). Quality and accuracy of electronic pre-anesthesia evaluation forms. Comput Methods Prog Biomed.

[21] Caban-Martinez AJ, Clarke TC, Davila EP, Fleming LE, Lee DJ (2011). Application of handheld devices to field research among underserved construction worker populations: a workplace health assessment pilot study. Environ Health.

[22] Dillon DG, Pirie F, Rice S, Pomilla C, Sandhu MS, Motala AA (2014). Open-source electronic data capture system offered increased accuracy and cost-effectiveness compared with paper methods in Africa. J Clin Epidemiol.

[23] Ebert JF, Huibers L, Christensen B, Christensen MB (2018). Paper- or web-based questionnaire invitations as a method for data collection: cross-sectional comparative study of differences in response rate, completeness of data, and financial cost. J Med Internet Res.

[24] Kongsved SM, Basnov M, Holm-Christensen K, Hjollund NH (2007). Response rate and completeness of questionnaires: a randomized study of internet versus paper-and-pencil versions. J Med Internet Res.

[25] Sebo P, Maisonneuve H, Cerutti B, Fournier JP, Senn N, Haller DM (2017). Rates, delays, and completeness of general practitioners’ responses to a postal versus web-based survey: a randomized trial. J Med Internet Res.

