# Correction: Staff preferences towards electronic data collection from a national take-home naloxone program: a cross-sectional study

**DOI:** 10.1186/s13011-024-00609-7

**Published:** 2024-05-19

**Authors:** Øystein Bruun Ericson, Desiree Eide, Philipp Lobmaier, Thomas Clausen

**Affiliations:** 1 Norwegian Centre for Addiction Research, Institute of Clinical Medicine, University of Oslo, P.O. box 1039 Blindern, Oslo, 0315 Norway; 2 King’s College London, National Addiction Centre, 4 Windsor Walk, London, SE58BB England

**Correction: Substance Abuse Treatment, Prevention, and Policy (2022) 17:13**

10.1186/s13011-022-00440-y

Following publication of the original article [1], we have been notified that affiliation 1 was published incorrectly.

It is now:

1 The Norwegian Centre for Addiction Research, Building 45, Ullevål Hospital, Kirkeveien 166, 0450 Oslo, Norway

It should be as per below:

1 Norwegian Centre for Addiction Research, Institute of Clinical Medicine, University of Oslo, P.O. box 1039 Blindern, Oslo 0315, Norway

